# Predominance of extensively-drug resistant *Acinetobacter baumannii* carrying *bla*_*OXA-23*_ in Jordanian patients admitted to the intensive care units

**DOI:** 10.1371/journal.pone.0317798

**Published:** 2025-02-27

**Authors:** Qutaiba Ababneh, Neda’a Aldaken, Ziad Jaradat, Ekhlas Al-Rousan, Zeina Inaya, Dua’a Alsaleh, Dua’a Alawneh, Sara Al Sbei, Ismail Saadoun

**Affiliations:** 1 Department of Biotechnology and Genetic Engineering, Faculty of Science and Arts, Jordan University of Science and Technology, Irbid, Jordan; 2 Department of Applied Biology, College of Science, University of Sharjah, Sharjah, United Arab Emirates; Shiraz University of Medical Sciences, IRAN, ISLAMIC REPUBLIC OF IRAN

## Abstract

**Background and Aim:**

The global emergence of *Acinetobacter baumannii* is of great concern, especially inside intensive care units (ICUs). This study investigated the prevalence, antibiotic resistance, biofilm formation and genetic relatedness of *A. baumannii* recovered from ICU patients in three major hospitals in Jordan.

**Methods:**

The *A. baumannii* isolates included in this study were identified by the detection of the *bla*_*OXA-51*_ gene, and a multiplex PCR assay. Antibiotic susceptibility testing was performed using the disk diffusion and broth microdilution methods, and the ability of the isolates to form biofilms was tested using the 96-well plate assay. All isolates were tested for the presence of carbapenemases-encoding genes by PCR. Clonal relatedness was assessed by Rep-PCR and dendrogram analysis.

**Results:**

Overall, 148 *A. baumannii* isolates were identified, with 96.7% of the isolates recognized as carbapenem resistant *A. baumannii*. Based on their resistance patterns, 90% of the isolates were extensively resistant (XDR). The highest prevalence of carbapenemases-encoding genes was for *bla*_*OXA-23-like*_ (96.7%), followed by *bla*_*ADC*_ (93.9.2%), *bla*_*VIM*_ (56.8%) and *bla*_*NDM-1*_ (7.4%). Almost 80% of the isolates were able to form biofilms, with 63.2% classified as strong biofilm former. Rep-PCR and clustering analysis revealed 26 different clusters and the circulation of hospital-specific clones.

**Conclusions:**

Our study revealed an alarming high prevalence of XDR, *bla*_*OXA-23*_-carrying and strong biofilm-producing *A. baumannii* among ICU patients. These findings call for continuous epidemiological surveillance and implementation of prevention strategies to reduce infections and dissemination of such a problematic pathogen inside the ICUs.

## Introduction

In hospitals and other healthcare settings, particularly inside ICUs, infections caused by multi-drug resistant bacteria are increasing at an alarming rate. ICUs are crucial for maintaining the lives of patients who are critically ill or unconscious. However, ICU patients are always at an increased risk of infections due to their delayed immune responses, and the use of invasive devices as part of their treatment, in addition to the usual long stay [1]. Although antibiotic treatments have reduced the deaths and illness of such infections, the extensive use of antibiotic has created a selective pressure that cleared susceptible bacteria, allowing the spread of resistant strains [2]. While carbapenems were once a cornerstone of treatment for critical infections, their extensive use has led to the emergence and spread of carbapenem-resistant pathogens in ICUs, posing a significant threat to patients [3,4]. Among these pathogens, carbapenem-resistant *A. baumannii* (CRAB) is one of the leading causes of healthcare-associated infections (HAIs) inside the ICUs [5]. For this reason, this bacterium made the World Health Organization’s (WHO) list of bacteria that pose the greatest threat to human health, and for which novel therapeutics are critically needed.

Although CRAB is mainly associated with respiratory tract infections in ICUs, particularly ventilator-associated pneumonia, it can also cause other types of infections such as bacteremia, secondary meningitis, as well as urinary tract, soft tissue, and wound infections [6]. CRAB infections have been shown to increase patient length and cost of ICU stay, as well as increase the use of antibiotics [7]. The prevalence and risk factors of CRAB infections have gained increasing attention due to the growing number of CRAB isolates found in patients. Currently, healthcare physicians are left with few effective therapeutic options to combat CRAB, with colistin and tigecycline being the last-resort agents used to treat infections caused by this pathogen, but their use is limited due to nephrotoxicity and neurotoxicity [8]. Unfortunately, CRAB strains resistant to these last-resort agents have been increasingly reported worldwide [9].

A growing body of research has been conducted on the antibiotic resistance mechanisms and genetic diversity of CRAB isolates from ICUs, with most studies carried out in Asia and Africa [10]. While the *bla*_*OXA-23-like*_ gene has been the dominant antibiotic resistance gene in most studies, variations in both antibiotic resistance genes and CRAB molecular types have been observed across different regions and countries. Thus, this study aimed to investigate the molecular epidemiology, antimicrobial susceptibility and biofilm formation ability of CRAB isolates recovered from ICU patients in hospitals serving the three largest governates in Jordan.

## Materials and methods

### Clinical isolates

Non-redundant, single-patient *A. baumannii* clinical isolates were collected from three major hospitals in the 3 largest populated governates in Jordan; King Abdullah University Hospital (KAUH), New Zarqa Governmental Hospital (ZH) and Al-Bashir Hospitals (BH). Permission to collect the isolates was obtained from the Jordan Ministry of Health (MOH) and the collection was performed according to the MOH regulations. All *A. baumannii* isolates chosen in this study were previously identified using the VITEK^©^2 (Compact workflows) system available in each hospital. Information collected for each isolate included: the hospital, type of clinical specimen, patient age and gender. The isolates were collected between January 2019 and February 2020 and were stored at − 80°C.

### Molecular identification of the isolates

Molecular identification of *A. baumannii* was conducted by PCR amplification of a segment of the *bla*_*OXA-51*_ gene [11]. Also, a multiplex PCR assay as described previously [12]. Genomic DNA was was performed to differentiate between different species of *Acinetobacter* extracted using the Quick-DNA Miniprep Plus (ZymoResearch, USA) following the manufacturer’s instructions. DNA from the reference strain *A. baumannii* ATCC 19606 was used as a positive control.

### Antimicrobial susceptibility testing

All antibiotic testing procedures were performed according to Clinical and Laboratory Standards Institute (CLSI) guideline [13]. Each isolate was tested against 19 different antibiotic discs belonging to 9 antibiotic classes. The Kirby-Bauer disc diffusion method was used to test the susceptibility of all isolates against the following antibiotics: Piperacillin/Tazobactam (TZP, 10 µg), Ertapenem (ETP, 10 µg), Tetracycline (TE, 30 µg), Ciprofloxacin (CIP, 5 µg), Trimethoprim/Sulfamethoxazole (SXT, 1.25/23.75 µg), Ampicillin/Sulbactam (SAM, 10 µg), Doripenem (DOR, 10 µg), Ceftriaxone (CRO, 30 µg), Levofloxacin (LEV, 5 µg), Ceftazidime (CAZ, 30 µg), Imipenem (IPM, 10 µg), Ampicillin (AMP, 10 µg), Meropenem (MEM, 10 µg), Norfloxacin (NOR, 10 µg) Cefepime (FEP, 30 µg), Tobramycin (TOB, 10 µg), Gentamicin (CN, 10 µg), Amikacin (AK, 30 µg) and Piperacillin (PRL, 100 µg). The minimal inhibitory concentrations (MICs) of the antimicrobial drug tigecycline, colistin and polymyxin B were determined by the broth microdilution method using a 96-well, flat bottom microtiter plates as described previously [14,15]. The *A. baumannii* ATCC BAA-1605 reference strain was used as positive control for all antimicrobial susceptibility testing.

### Biofilm formation

The crystal violet biofilm formation assay was performed using the semiquantitative method described by Hu et al (2016) [16]. Each isolate was assayed in triplicate at three independent time-points using fresh samples each time. The biofilm phenotype was determined for each isolate as described previously [17]. *Acinetobacter baumannii* ATCC 19606 was used as a positive control.

### PCR amplification of carbapenemase genes

Isolates were tested for the presence of the genes encoding the Ambler class B enzymes (*bla*_*IMP-type*_*, bla*_*VIM-type*_) and class D enzymes (*bla*_*OXA-23-like*_*, bla*_*OXA-24-like*_*, bla*_*OXA-51-like*_ and *bla*_*OXA-58-like*_) by PCR as described previously [18]. PCR products were purified and subjected to dideoxy chain termination sequencing (Macrogen, South Korea) for confirmation.

### Rep-PCR analysis and dendrogram

Rep-PCR typing was performed on all isolates as described previously [19]. The PCR conditions were as follows: an initial denaturation at 95°C for 3 min, then 30 cycles of denaturation at 90°C for 30 s, annealing at 45°C for 1 min, and extension at 65°C for 8 min, followed by a final extension at 65°C for 16 min. The PCR products were subjected to electrophoresis in 1.5% agarose gel. The patterns of REP bands of the 148 *A. baumannii* isolates were scored manually, with the data coded as a factor of 0 or 1, representing the absence or presence of bands, respectively. Using SPSS software, version 29.0, a dendrogram showing the genetic relatedness between the isolates was constructed using the Jaccard coefficient, and the clusters for the dendrogram were selected based on the similarity above 95%.

## Results

### Collection of samples and molecular identification of *A. baumannii* isolates

In this study, 152 single-patient, non-redundant *A. baumannii* isolates were recovered from clinical specimens of ICU patients admitted in 3 major hospitals in Jordan. These isolates were previously identified biochemically using the VITEK©2 Compact Workflow. The collection of isolates was conducted in strict accordance with ethical standards and was approved by the Institutional Review Board (IRB) at Jordan University of Science and technology (IRB no.14/111/2017). The IRB waived the need for consent since only data about the patients’ age and sex, type of infection, and place of hospitalization were collected, while patient identifying information were completely removed. To confirm the identity of the isolates at the species level, PCR amplification of a 545 bp region of the intrinsic *bla*_*OXA-51*_ gene and a multiplex PCR assay were used for this purpose. Following PCR, 148 single-patient, non-redundant *A. baumannii* isolates were included in this study. The patients’ male to female ratio from which the isolates were recovered is 1.77:1, with 64% and 36% of the isolates obtained from males and females, respectively. Table 1 summarizes the demographics and source data of all isolates investigated in the current study. About 61% of these isolates (n = 90) were collected from Al-Basheer hospitals (BH) in Amman city, which is the largest hospital in Amman, Jordan. The second-largest number of isolates were from King Abdullah University Hospital (KAUH) in northern Jordan (n = 39; 26%), while 13% of isolates (n = 19) were from the New Zarqa Governmental Hospital (ZH) located in Al-Zarqa city. Regarding the type of specimens from which the isolates were recovered, 60% were from sputum. The number of isolates from other types of specimens were as follows: urine (n = 6), blood (n = 16), cerebrospinal fluid (n = 12) and pus (n = 8).

**Table 1:** Summary of the demographic and source data of *A. baumannii* isolates included in this study.

Data Category	Hospital			Total number of isolates
	BH (n = 90)	KAUH (n = 39)	ZH (n = 19)	
Gender				
Male	**58**	**25**	**11**	**94**
Female	**32**	**14**	**8**	**54**
Type of clinical specimen				
Sputum	**47**	**34**	**12**	**91**
Urine	**4**	**2**	**0**	**6**
Blood	**14**	**2**	**0**	**16**
Cerebrospinal fluid	**11**	**0**	**1**	**12**
Pus	**5**	**0**	**1**	**6**
Others*	**11**	**1**	**5**	**17**

### Antibiotic susceptibility of the *A. baumannii* isolates

**Fig 1:**
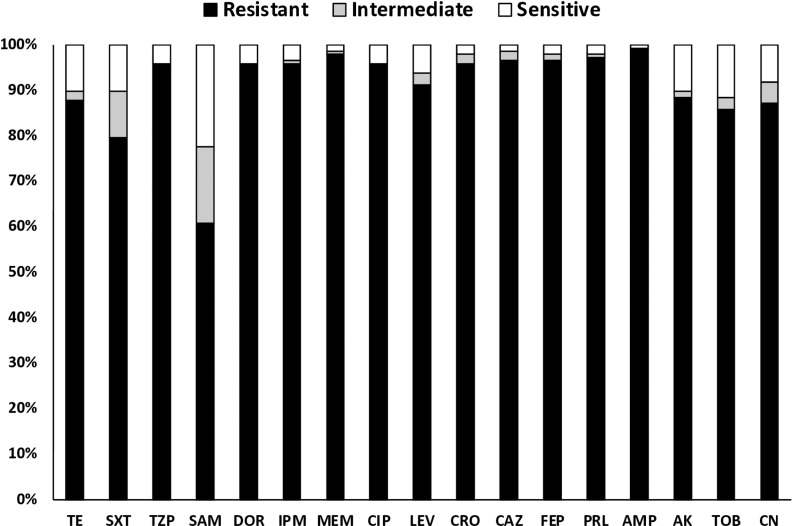
Percentages of the resistance phenotypes in 148 *A. baumannii* isolates for 17 antibiotics. Tetracycline (TE), Trimethoprim/Sulfamethoxazole (SXT) Piperacillin/Tazobactam (TZP), Ampicillin/Sulbactam (SAM), Doripenem (DOR), Imipenem (IPM), Meropenem (MEM), Ciprofloxacin (CIP), Levofloxacin.

Among the 148 *A. baumannii* isolates, between 95.9 to 96.7% were phenotypically resistant to 3 carbapenems; meropenem, doripenem and imipenem. Resistance to non-carbapenem beta-lactams, including those with a beta-lactamase inhibitor, ranged between 60.8% to 97.2%. The highest antibiotic resistance obtained was for ampicillin, with 99.3% of the isolates exhibiting resistance against this antibiotic. The percentages of resistant *A. baumannii* isolates to other antibiotics were as follows; aminoglycosides (85.8–88.8%), fluoroquinolones (91.2-95.9%), folate pathway antagonist (79.7%), and tetracyclines (87.8%). Fig 1 shows the percentages of the resistant, intermediately susceptible, and susceptible phenotypes for all antibiotics tested. The lowest levels of resistance were to tigecycline and polymyxins, with 3.4% and 1.4% of isolates being resistant to these agents, respectively.

### Distribution of resistant isolates according to the hospital source, gender, and the type of clinical specimen.

**Fig 2:**
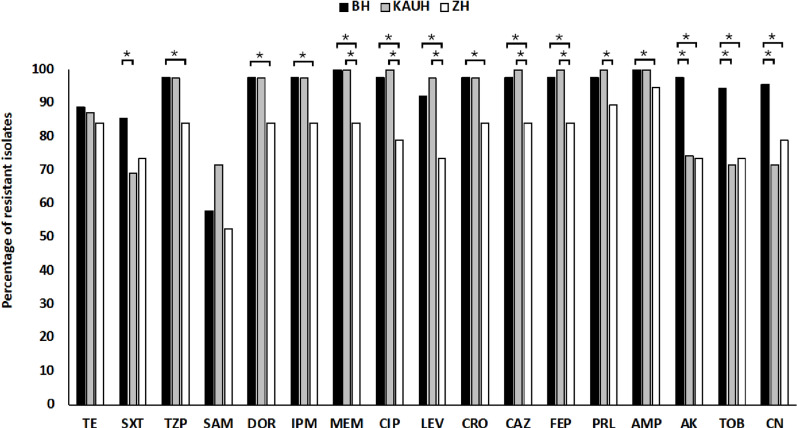
Distribution of *A. baumannii* resistant isolates according to the hospital source. KAUH: King Abdullah University Hospital, ZH: New Zarqa Governmental Hospital, BH: Al-basher Hospital. * : significant difference in the proportions of resistance, **P** < 0.05. Tetracycline (TE), Trimethoprim/Sulfamethoxazole (SXT) Piperacillin/Tazobactam (TZP), Ampicillin/Sulbactam (SAM), Doripenem (DOR), Imipenem (IPM), Meropenem (MEM), Ciprofloxacin (CIP), Levofloxacin (LEV), Ceftriaxone (CRO), Ceftazidime (CAZ), Cefepime (FEP), Piperacillin (PRL), Ampicillin (AMP), Amikacin (AK), Tobramycin (TOB), Gentamicin (CN).

The percentages of the resistance phenotypes for all isolates according to the hospital source were compared (Fig 2). Using the Chi-square test of proportions, we found a significant difference (p < 0.05) in the level of resistance between the isolates collected from KAUH and BH for gentamycin, tobramycin, amikacin and Trimethoprim/Sulfamethoxazole. In addition, a significant difference (p < 0.05) was observed in the level of resistance against meropenem, 2 fluoroquinolones, 3 cephems and piperacillin between the isolates originating from KAUH and ZH, as well as the isolates from BH and ZH. Lastly, a significant difference (p < 0.05) was found between the BH and ZH isolates in the level of resistance against 3 carbapenems, 3 cephems, 2 fluoroquinolones, gentamycin, tobramycin, ampicillin, amikacin and piperacillin/tazobactam.

**Fig 3:**
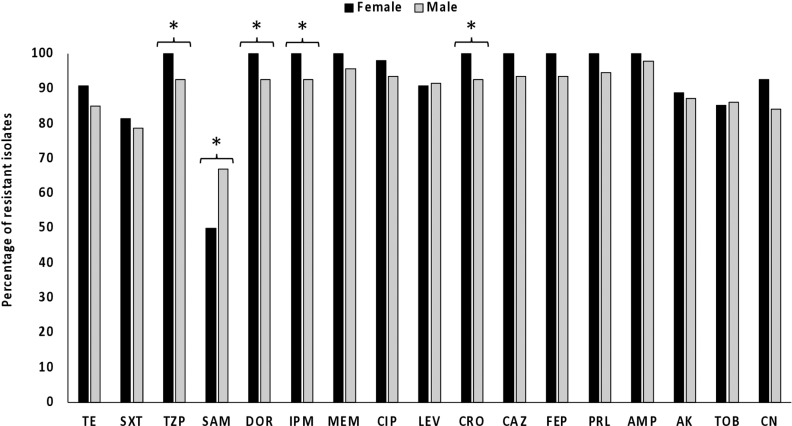
Distribution of the resistant *A. baumannii* isolates according to the patient’s gender. * : significant difference in the proportions of resistance, **P** < 0.05. Tetracycline (TE), Trimethoprim/Sulfamethoxazole (SXT) Piperacillin/Tazobactam (TZP), Ampicillin/Sulbactam (SAM), Doripenem (DOR), Imipenem (IPM), Meropenem (MEM), Ciprofloxacin (CIP), Levofloxacin (LEV), Ceftriaxone (CRO), Ceftazidime (CAZ), Cefepime (FEP), Piperacillin (PRL), Ampicillin (AMP), Amikacin (AK), Tobramycin (TOB), Gentamicin (CN).

The average percentage of the resistance phenotype for all antibiotics for the female and male isolates was 70%. Comparison of the level of resistance between the male and females isolates revealed significant differences for 5 antibiotics, piperacillin/tazobactam, ampicillin/sulbactam, doripenem, imipenem and ceftriaxone (Fig 3).

### Classification of the isolates according to their resistance patterns

Based on the antibiotic resistance patterns, the isolates were classified into three phenotypes, non-multidrug-resistant (non-MDR), multidrug-resistant (MDR) and extensively drug-resistant (XDR). A total of 133 (90%) isolates were XDR, 12 isolates were MDR (8.1%) and only 3 (2%) were non-MDR isolates (Table 2). The highest proportion of XDR isolates was collected from Al-Bashir hospital (96.7%, 87/90), followed by New Zarqa Governmental Hospital (84.2%, 16/19) and KAUH (76.9%, 30/39). With regard to the type of clinical specimens, all the 12 isolates recovered from CSF samples were classified as XDR, while the proportion of XDR isolates from the other types of samples were as follows: blood (87.5%, 14/16), sputum (87.9%, 80/91), pus (83.3%, 5/6) and urine (83.3%, 5/6). Table 2 shows the distribution of classified isolates according to hospital source, gender and type of clinical specimen.

**Table 2:** Distribution of resistance patterns according to hospital source, gender and type of clinical specimen.

Factor	Percentages of resistance pattern		
	Non-MDR	MDR	XDR
**Hospital**			
Al-Bashir Hospital (n = 90)	1.1%	2.2%	96.7%
King Abdullah University Hospital (n = 39)	0%	23.1%	76.9%
New Zarqa Governmental Hospital (n = 19)	5.3%	10.5%	84.2%
**Gender**			
Female (n = 54)	0%	7.4%	92.6%
Male (n = 94)	3.2%	8.5%	88.3%
**Type of clinical specimen**			
Urine (n = 6)	16.7%	0%	83.3%
Pus (n = 6)	16.7%	0%	83.3%
Sputum (n = 91)	0%	12.1%	87.9%
Blood (n = 16)	6.3%	6.3%	87.5%
Cerebrospinal Fluid (n = 12)	0%	0%	100%
Others * (n = 17)	0%	0%	100%

### Screening of β-lactam resistance genes

DNA samples extracted from 148 isolates were screened for the presence of 11 β-lactam resistance genes (*bla* genes). None of the isolates tested positive for the presence of *bla*_OXA58-Like_, *bla*_OXA24-Like_ *bla*_OXA143-Like_, *bla*_IMP_, *bla*_GIM_, *bla*_SPM_, and *bla*_SIM_. The incidence of other invstigated resistance genes among all isolates was as follows: 143 (96.7%) isolates harbored *bla*_OXA23-Like_, 139 (93.9%) isolates harbored *bla*_ADC_, 85 (56.8%) isolates harbored *bla*_VIM_, and 11 (7.4%) isolates harbored *bla*_NDM_.

### Biofilm formation

**Fig 4:**
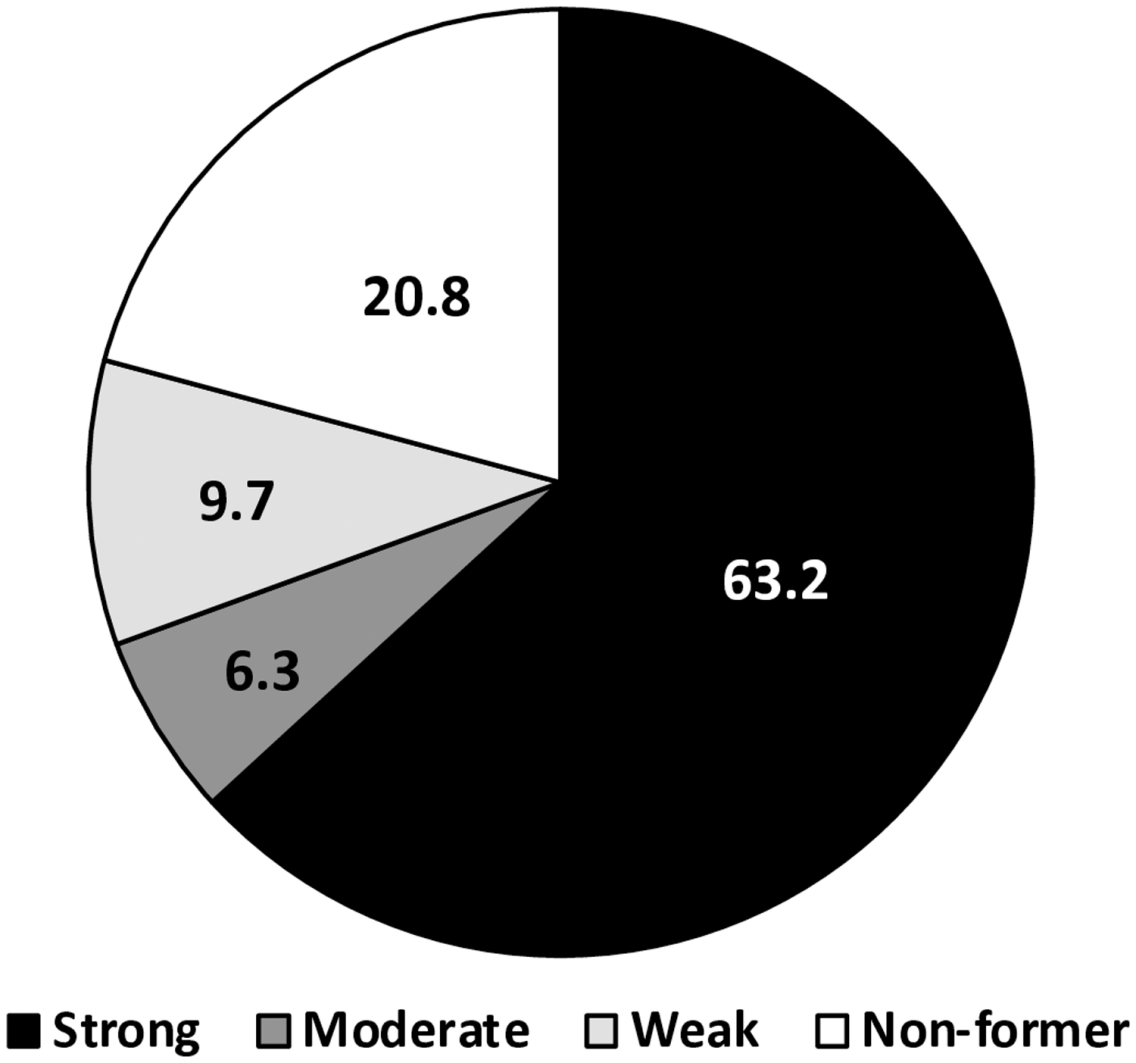
Distribution of *A. baumannii* isolates according to the biofilm formation phenotype.

The ability to form biofilms was tested for all isolates in triplicates (3 independent days) and standard errors were calculated to classifying each isolate into one of the four biofilm formation phenotypes; non-former, weak, moderate and strong former. The majority of the isolates were able to form biofilm (79.8%) with varying abilities, where 63.2% were strong biofilm formers, while 9.7% and 6.3 of the isolates showed moderate and weak ability to biofilm formation, respectively (Fig 4). With respect to the hospital source of the isolates, the prevalence of strong biofilm formers was the highest among the isolates from BH (78.2%), followed by ZH (68.4%) and KAUH (28.2%)

### Clonal relatedness of the isolates

**Fig 5:**
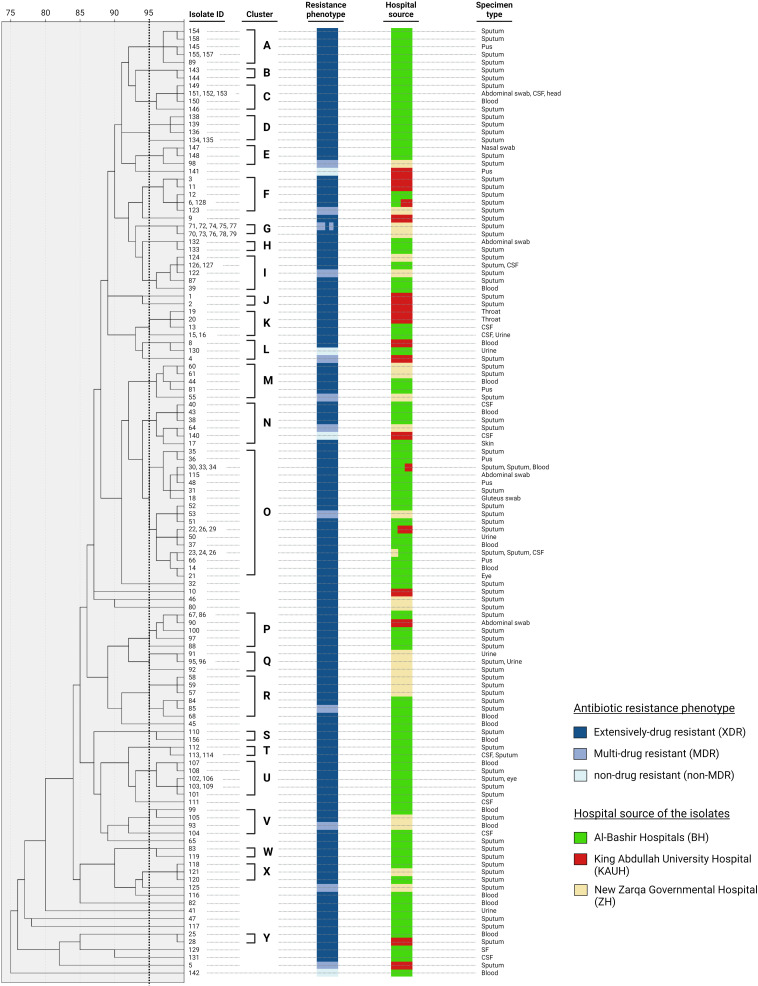
Dendrogram based on rep-PCR fingerprint analysis of *A. baumannii* isolates. For the purpose of predicting different clusters, the top match feature at > 95% similarity was used.

The clonal relatedness of the 148 isolates was invistigated by repPCR and dendrogram analysis. The similarity of the tested isolates ranged from 75 to 99%. Overall, the dendrogram analysis revealed 25 different clusters (A-Y) and 18 singletons (Fig 5). Clusters A, B, C, D, H, S, T, U, and W, which contained 36 (24.3%) isolates, comprised of XDR isolates originated from AlBasheer hospitals, and were mainly recovered from sputum specimens. The largest cluster O contained 23 isolates, with most isolates were XDR and originated from AlBasheer hospitals. Isolates in clusters G (n = 10) and Q (n = 14) originated from New Zarqa Governmental hospital with being mainly recovered from sputum specimens. Clusters F, J, K contained 13 (8.7%) XDR isolates, with 7 of these isolates originating from KAUH. The remaining clusters showed diversity in the resistance profiles of their isolates, as well as their hospital and specimen sources.

## Discussion

*A. baumannii* has become a critical threat worldwide due to its ability to survive in hospital environments and cause frequent nosocomial outbreaks. In this study we characterized 148 *A. baumannii* isolates recovered from patients admitted to ICUs in 3 different hospitals serving the three largest cities in Jordan. More than 60% of the isolates were recovered from clinical specimens of respiratory origin. The respiratory tract is the most common isolation site of *A. baumannii* isolates in ICU patients [20], which is attributed to the critical condition of these patients, as well as to certain medical procedures such as tracheostomy, trachea intubation, mechanical ventilation, and sputum aspiration. Such procedures can damage the mucous epithelia of the respiratory tract, increasing the chance for *A. baumannii* to adhere and colonize the respiratory tract [21]. We noticed a sex-based difference in the number of *A. baumannii* isolated from male and female patients, with almost two thirds of the isolates recovered from males, which is in accordance with the findings of previous studies [22–24]. Sex bias towards certain types of infectious diseases have been reported previously. For example, females are more susceptible to HIV, malaria and *Legionella pneumophila* infections than men [25]. The sex bias observed in our study could be explained by differences in the genetic makeup, and sex hormones between males and females. In addition, men in our country are generally more active than women, and thus are more likely to be exposed to environments contaminated with bacteria. Previous results attributed this sex bias to differences in the antibiotic prescribed to males and females [26], or lower adherence among men to hand hygiene guidelines [27]. Our results highlight the importance of implementing surveillance procedures and infection control measures that take into consideration the sex of the ICU patients. By the same token, doctors should take into account that males are a risk group of *A. baumannnii* infections when considering empiric antibiotic treatment.

Testing the isolates for antimicrobial susceptibility revealed a concerning high level of resistance against carbapenems, with 96.7% of isolates categorized as CRAB, which agrees with the resistance levels reported by previous studies [23,28,29]. We noticed that the resistance to meropenem is slightly higher compared to imipenem. This finding has been demonstrated by many other studies [30]. Carbapenems are considered second-line agents for treatment of *A. baumannii* infections. Therefore, this high level of carbapenem resistance limits the number of antimicrobial agents available for treatment, which makes it difficult to treat CRAB infections, and might increase the cost of hospitalization and increase mortality rates among ICU patients. Factors that might contribute to the increase in the incident of CRAB isolates include, inadequate infection control measures, imprudent use of carbapenems to treat CRAB infections in the ICUs and poor management of antimicrobial stewardship.

The CRAB isolates exhibited high resistance rates to aminoglycosides, cephems, fluoroquinolones, β-Lactam combination agents, penicillins, and tetracycline, which ranged between 73% to 91%. Therefore, most of these isolates were classified as XDR. This resistance trend is consistent with the findings reported by Xie *et al* (2018), who in their study performed a systematic review and meta-analysis of the global prevalence of resistance in *A. baumannii* to commonly prescribed antibiotics. It was concluded in this study that the rate of antibiotic resistance has increased in recent years [31]. On the other hand, the majority of isolates exhibited high susceptibility (93%) to tigecycline and colistin, which is consistent with several ptrvious reports [32–34]. These antibiotics are practically important for treatment of carbapenem-resistant *A. baumannii* infections, either alone or in combination with other antimicrobial agents [33,35]. Still, the high incidence of XDR isolates in this study is alarming, as these isolates are at risk of becoming pandrug resistant, potentially limiting the treatment options for infected patients. In Jordan, previous studies reported the isolation of XDR *A. baumannii* isolates from clinical specimens collected from non-ICU patients [23,36], both hospital and community environmental samples [37,38], fresh produce [39] and even spices and herbs [40].

The resistance incidence of the *A. baumannii* isolates was compared according to the patient’s sex and the source of isolation from the hospital. A significant difference was observed in the resistance incidence of isolates recoverd from males and females for 5 out of the 17 tested antibiotics. Similar findings were reported by another recent study [41]. Although the underlying mechanisms of this sex bias is still unclear, it may be explained by differences in the antibiotics prescribed to males and female. It is also possible that the higher *A. baumannii* infection rate of males is the reason behind this sex bias towards these antibiotics and not the antibiotics themselves. The resistance profiles for 15 antibiotics significantly differed according to the hospital from which the isolates were collected, with BH (n = 90) and KAUH (n = 39) isolates exhibiting a higher resistance rates compared to the ZH isolates (n = 19). This significant difference could be due to the low number of ZH isolates investigated, the types of antibiotics prescribed to ICU patients in this hospital, or other local factors. Also, ZH is the newest hospital and the smallest among the 3 hospitals included in this study, and therefore the *A. baumannii* strains detected from this hospital among ICU patients might still being introduced from outside the hospital. In addition, these strains are yet to accumulate antibiotic resistance determinants similar to those found in the strains recovered from the other two hospitals which were circulating around for longer times. Nonetheless, antibiotic treatment for ICU patients can be more effective if the data about single-hospital prevalence, risk factors, and the antibiotic susceptibility of *A. baumannii* are taken into consideration.

A*. baumannii* is known for its ability to colonize and form biofilms [42]. Biofilms provide protection for microbes against antimicrobial agents, host immune defense and harsh environmental conditions [43]. Additionally, antibiotic resistance of bacteria in such protected communities can be increased up to 1000- fold [44] due to the conditions that favor the exchange of resistance genetic determinants between members of the biofilm community. In the present study, almost 80% of the isolates demonstrated the ability to form biofilm *in vitro*, with 63.2% of these isolates displaying a strong ability to form biofilms. Moreover, the majority of the strong biofilm formers (92.3%) exhibited the XDR resistance pattern, highlighting a concerning association between biofilm formation and antibiotic resistance. Previous studies have reported an increased biocidal and antimicrobial resistance among the strong *A. baumannii* biofilm formers, suggesting that biofilm formation is an important factor in the persistence of *A. baumannii* inside the hospital environments [43]. In addition, biofilms can extend the survival duration and desiccation tolerance of *A. baumannii*. The high incidence of XDR and strong biofilm-formering *A. baumannii* poses a huge risk to ICU patients and staff, as these isolates can potentially form surface biofilms that persist for long periods and transmit pathogens and antibiotic resistance determinants. The ICU contains several types of inanimate surfaces that have been previously shown to harbor biofilms, such as bed rails, food tables, medical devices, sinks and many others [38,45–47].

*A. baumannii* is a successful nosocomial pathogen inside the ICUs, therefore investigating the genetic relatedness of the isolates recovered from ICU patients is important to develop effective strategies to prevent their spread [48]. We utilized rep-PCR to infer the degree of clonal relatedness of the *A. baumannii* isolates investigated in this study. This method offers a comparable discriminatory power to pulse filed gel electrophoresis PFGE [49,50]. The isolates were classified into 25 different clonal clusters, A to Y, according to banding pattern obtained by Rep-PCR. A number of closely related isolates (n = 19), which clustered into 4 clusters (A-D), were all recovered from Albasheer hospital only. These isolates are XDR, strong biofilm former, and harboring the *bla*_*OXA-23*_ gene. This striking similarity might indicate that these strains originated from an endemic clone. The dendrogram also revealed another 12 closely related isolates belonging to 3 clusters (S-U). This suggests that at least two clones and their closely related strains are circulating and causing infections to ICU patients in Albasheer. Interestingly, cluster G comprised 10 isolates recovered from AlZarqa hospital. Four of these isolates tested positive for the *bla*_*NDM-1*_ gene, generated identical banding pattern in Rep-PCR and carried the *bla*_*NDM-1*_ gene, indicating a *bla*_*NDM-1*_*-*carrying strain that is endemic in this hospital.

In conclusion, this study highlights the high incidence of XDR, *bla*_*OXA-23*_-carrying and strong biofilm-producing *A. baumannii* among ICU patients in 3 major hospitals in Jordan. The ability of these isolates to form strong biofilms further complicates treatment and infection control efforts. The study also emphasized the sex-based difference in the number of *A. baumannii* isolates, highlighting the importance of considering the sex of the ICU patients. Our findings underscore the need for continuous epidemiological and microbiological surveillance, and the implementation of prevention strategies aimed at reducing infections and dissemination of such a problematic pathogen inside the ICUs.

## Supporting information

S1 Data Characteristics of all isolates included in the study. (XLSX)
